# Spatiotemporal Correlations between Cytosolic and Mitochondrial Ca^2+^ Signals Using a Novel Red-Shifted Mitochondrial Targeted Cameleon

**DOI:** 10.1371/journal.pone.0045917

**Published:** 2012-09-21

**Authors:** Markus Waldeck-Weiermair, Muhammad Rizwan Alam, Muhammad Jadoon Khan, Andras T. Deak, Neelanjan Vishnu, Felix Karsten, Hiromi Imamura, Wolfgang F. Graier, Roland Malli

**Affiliations:** 1 Institute of Molecular Biology and Biochemistry, Centre of Molecular Medicine, Medical University of Graz, Graz, Austria; 2 Precursory Research for Embryonic Science, Japan Science and Technology Agency, Tokyo, Japan; University of California, Berkeley, United States of America

## Abstract

The transfer of Ca^2+^ from the cytosol into the lumen of mitochondria is a crucial process that impacts cell signaling in multiple ways. Cytosolic Ca^2+^ ([Ca^2+^]_cyto_) can be excellently quantified with the ratiometric Ca^2+^ probe fura-2, while genetically encoded Förster resonance energy transfer (FRET)-based fluorescent Ca^2+^ sensors, the cameleons, are efficiently used to specifically measure Ca^2+^ within organelles. However, because of a significant overlap of the fura-2 emission with the spectra of the cyan and yellow fluorescent protein of most of the existing cameleons, the measurement of fura-2 and cameleons within one given cell is a complex task. In this study, we introduce a novel approach to simultaneously assess [Ca^2+^]_cyto_ and mitochondrial Ca^2+^ ([Ca^2+^]_mito_) signals at the single cell level. In order to eliminate the spectral overlap we developed a novel red-shifted cameleon, D1GO-Cam, in which the green and orange fluorescent proteins were used as the FRET pair. This ratiometric Ca^2+^ probe could be successfully targeted to mitochondria and was suitable to be used simultaneously with fura-2 to correlate [Ca^2+^]_cyto_ and [Ca^2+^]_mito_ within same individual cells. Our data indicate that depending on the kinetics of [Ca^2+^]_cyto_ rises there is a significant lag between onset of [Ca^2+^]_cyto_ and [Ca^2+^]_mito_ signals, pointing to a certain threshold of [Ca^2+^]_cyto_ necessary to activate mitochondrial Ca^2+^ uptake. The temporal correlation between [Ca^2+^]_mito_ and [Ca^2+^]_cyto_ as well as the efficiency of the transfer of Ca^2+^ from the cytosol into mitochondria varies between different cell types. Moreover, slow mitochondrial Ca^2+^ extrusion and a desensitization of mitochondrial Ca^2+^ uptake cause a clear difference in patterns of mitochondrial and cytosolic Ca^2+^ oscillations of pancreatic *beta-*cells in response to D-glucose.

## Introduction

Mitochondrial uptake of Ca^2+^ has been identified as a crucial process in cell physiology [1], [2]. Basically, mitochondria respond to Ca^2+^ and compete for Ca^2+^ with other organelles within a cell. A rise of the Ca^2+^ concentration in the lumen of mitochondria stimulates ATP generation [3], [4], which is important to compensate for increased ATP demands during cell stimulation [5]. Because of the high capacity of respiring mitochondria to sequester Ca^2+^, these organelles have also been recognized to locally clear cytosolic Ca^2+^ elevations [6]. This mitochondrial Ca^2+^ buffer function impacts the spatiotemporal pattern of cellular Ca^2+^ signals and, thus, significantly influences Ca^2+^ sensitive processes within a cell [7]–[10]. Besides the vital aspects of mitochondrial Ca^2+^ signaling, excessive mitochondrial Ca^2+^ loads trigger cell death [5]. Accordingly, the mitochondrial Ca^2+^ homeostasis can determine life and death of a cell [11], which points to its central role in cell physiology [12].

The investigation of mitochondrial Ca^2+^ signals in intact cells requires sophisticated methods to specifically quantify changes of the free mitochondrial Ca^2+^ concentration ([Ca^2+^]_mito_) [13]. One important property of a mitochondrial Ca^2+^ indicator is its correct targeting to the mitochondrial matrix. Out of many different chemical fluorescent Ca^2+^ indicators, only the positively charged dye Rhod-2 accumulates within respiring mitochondria [14]. However, Rhod-2 is not a ratiometric Ca^2+^ sensor making it difficult to quantify Ca^2+^ signals and under most conditions significant amounts of Rhod-2 is also found in the cytosol and other cellular compartments. In contrast, genetically encoded Ca^2+^ indicators that additionally contain mitochondria-targeting peptides are selectively targeted to the mitochondrial matrix [13]. Initially, the Ca^2+^ sensing bioluminescent protein aequorin was successfully targeted to mitochondria, which was primarily used to demonstrate that mitochondria definitely sequester Ca^2+^ under physiological conditions of cell stimulation [15]. Because of the weak signals from the bioluminescent reaction, the aequorin-based probes can hardly be used to monitor [Ca^2+^]_mito_ on the single (sub)cellular level. This limitation does not persist for the genetically encoded fluorescent mitochondrial Ca^2+^ sensors. Principally, these Ca^2+^ indicators, which consist of one or two fluorescent proteins (FPs), sense Ca^2+^ via a calmodulin (CaM) domain and the Ca^2+^-CaM interacting peptide from the myosin light chain kinase, M13 [16]. Binding of Ca^2+^ results in conformational changes of these domains, which alter the spectral properties of the adjacent FPs and accordingly [Ca^2+^]_mito_ can be assessed directly [17]. One of the genetically encoded fluorescent Ca^2+^ sensors that is frequently used to measure [Ca^2+^]_mito_ is the mitochondrial targeted ratiometric pericam (mtRP). The mtRP consists of a circularly permutated yellow FP that is flanked by CaM and M13 [18]. The mtRP is, however, highly sensitive to changes of the pH if the probe is excited at 480 nm, which considerably affects the fluorescence signal that increases upon binding of Ca^2+^ [19], [20]. Another group of genetically encoded fluorescent Ca^2+^ sensors are the so-called cameleons. In the cameleons the CaM and the M13 peptide are fused in tandem between a cyan fluorescent protein (CFP) and a yellow fluorescent protein (YFP). Binding of Ca^2+^ to cameleons narrows the distance between FPs yielding an increase of the Förster resonance energy transfer (FRET) from CFP (FRET-donor) to YFP (FRET-acceptor) [17]. The suitability of these FRET-based ratiometric Ca^2+^ indicators were further improved by engineering CaM and M13, which minimized the interaction with endogenous CaM and increased the range of the measurable Ca^2+^ concentrations [21].

For the spatiotemporal correlation between [Ca^2+^]_cyto_ and [Ca^2+^]_mito_ the measurement of both signals within same individual cells is desirable. However, co-imaging of most of the cytosolic fluorescent chemical Ca^2+^ indicators with genetically encoded fluorescent Ca^2+^ probes is problematic due to spectral overlaps of the excitation and/or emission wavelengths [22]. In some studies it has been reported that the cross-talk between the most widely used ratiometric cytosolic Ca^2+^ dye, fura-2/AM and CFP/YFP-based cameleons can be minimized by narrowing the bandwidth of emission filters [22]. However, as a result the intensities of the respective fluorescence signals of both Ca^2+^ indicators are reduced, yielding poor signal-to-noise ratios.

In this study we developed a novel red-shifted cameleon, which we named D1GO-Cam. In comparison to CFP/YFP-based cameleons the spectral crosstalk between fura-2 and D1GO-Cam was significantly diminished. D1GO-Cam could be targeted successfully to mitochondria and was used together with fura-2 to co-image [Ca^2+^]_mito_ and [Ca^2+^]_cyto_ within same individual cells. Applying simultaneous cytosolic and mitochondrial Ca^2+^ measurements using fura-2 and the novel mitochondria-targeted D1GO-Cam, respectively, a significant lag between increase of [Ca^2+^]_cyto_ and [Ca^2+^]_mito_, could be demonstrated, which depended on the mode of Ca^2+^ mobilization and the cell type used. Furthermore, a slow mitochondrial Ca^2+^ extrusion and a desensitization of mitochondrial Ca^2+^ uptake counteracted the synchronous transfer of cytosolic Ca^2+^ oscillations into mitochondria of pancreatic *beta-*cells, thus, partially uncoupling mitochondrial Ca^2+^ signal from cytosolic Ca^2+^ spiking.

## Results

### Development of Red-Shifted Cameleons

**Figure 1:**
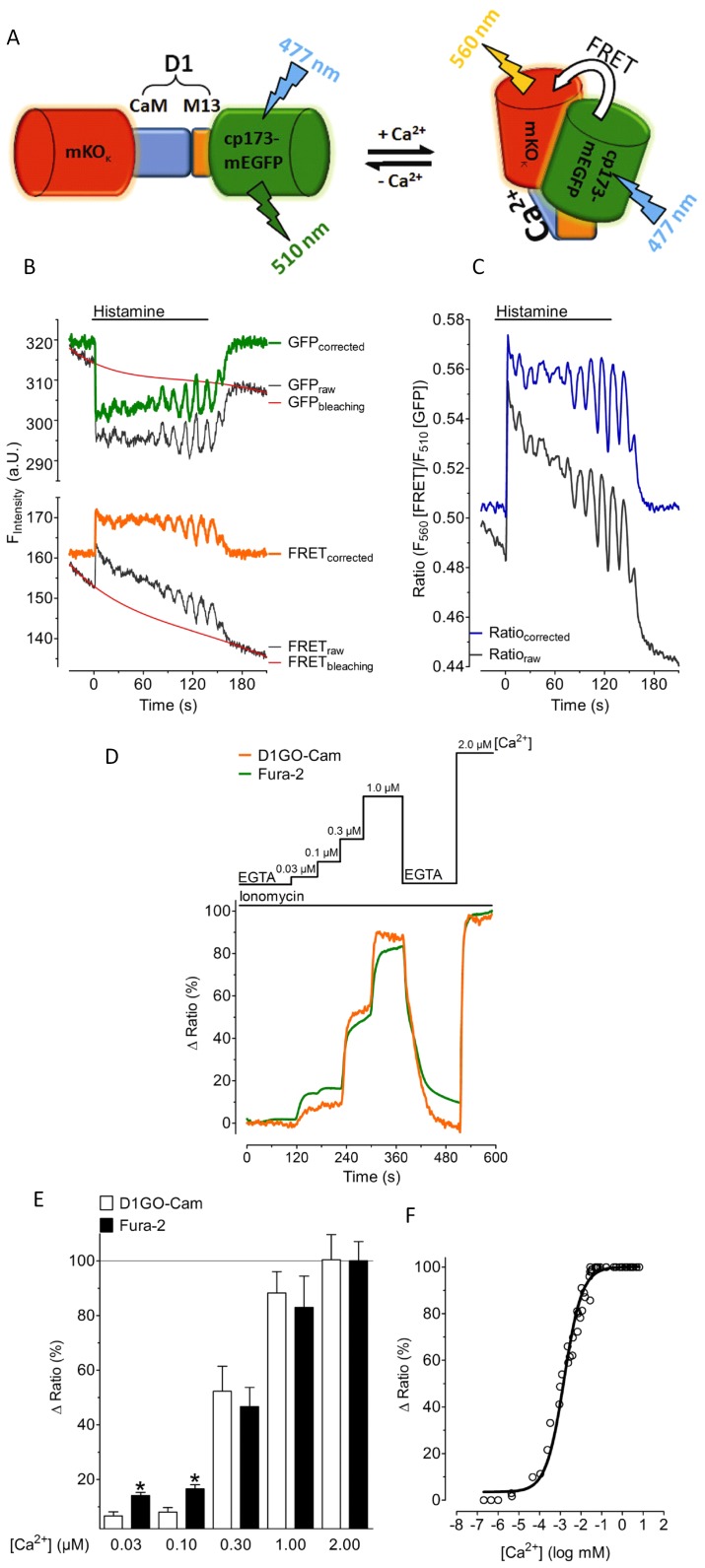
Characterization of D1GO-Cam. (***A***) Schematic representation of D1GO-Cam. D1GO-Cam consists of the orange fluorescence protein, (mKO_κ_) as the FRET acceptor and a circular permutated green fluorescent protein (cp173-mEGFP) as the FRET donor. In contrast to existing cameleons the FRET acceptor (mKO_κ_) is on the N-terminus in front of the Ca^2+^ sensitive domain, while the FRET donor (cp173-mEGFP) is on the C-terminus. FRET between cp173-mEGFP and mKO_κ_ is increasing upon binding of Ca^2+^ to approved CaM/M13 sequences of the design 1 (D1) of D1GO-Cam. The flash symbols indicate optimal excitation and emission wavelength for imaging GO-Cams. (***B***) Representative recordings of cytosolic Ca^2+^ oscillations upon cell stimulation with 100 µM histamine in intact HeLa cells expressing D1GO-Cam. The black curves represent original traces of the FRET donor GFP (upper panel) and the respective FRET signal (lower panel). Original traces (black) were corrected for photobleaching using the red curves (representing the bleaching functions regarding a one phase exponential decay) yielding the corrected traces for the GFP (FRET donor, green trace, upper panel) and the FRET channel (orange trace, lower panel), respectively. (***C***) Ratios of FRET/GFP of raw and corrected values from panel B. (***D***) Representative tracings of cytosolic Ca^2+^ signals measured with either D1GO-Cam (orange curve) or fura-2 (green curve) in ionomycine (3 µM) treated HeLa cells at various Ca^2+^ concentrations. (***E***) Comparative statistics of delta maximal ratios in percentage of D1GO-Cam (white column, n = 8) and fura-2 (black column, n = 8) at different Ca^2+^ concentrations ([Ca^2+^]) in ionomycin (3 µM) treated HeLa cells. The average of delta maximal ratios at 2.0 µM Ca^2+^ were defined as 100%. (***F***) The *in situ* Ca^2+^ concentration response curve of D1GO-Cam was calculated from experiments using ionomycin (10 µM) treated HeLa cells. The actual Ca^2+^ concentrations plotted were determined using respective fura-2 or Magfura-2 signals, which were recorded simultaneously. The curve shown here represents an average of 7 independent experiments.

In analogy to a recently developed red-shifted FRET-based ATP probe [23] the CFP and YFP of cameleons were replaced by the OFP variant, mKO_κ_, and a circularly permutated green fluorescent protein, (cp173-mEGFP), respectively. We named this red-shifted cameleon “green-orange cameleon” (GO-Cam). The whole Ca^2+^ sensitive designed calmodulin/M13 sequence (D1) [21] from the D1ER probe [24], [25] was fused in tandem between the mKO_κ_ and cp173-mEGFP yielding a genetically encoded Ca^2+^ probe, which we termed D1GO-Cam (Figures 1A).

### Characterization of D1GO-Cam

First, we tested D1GO-Cam in intact HeLa cells. In all measured cells the donor fluorescence of D1GO-Cam was approximately 2 fold higher than the respective FRET signal under resting conditions (cp173-mEGFP fluorescence intensity was 275.9±41.8, versus a FRET signal intensity of 133.8±15.3, n = 23, Figure 1B). The cp173-mEGFP (FRET donor) of D1GO-Cam was illuminated with 477 nm and both the donor emission and the FRET signal (FRET to the acceptor mKO_κ_ emission) were quantified simultaneously using a beam splitter device. The fluorescence intensities in the cp173-mEGFP and FRET channels continuously declined with slow kinetics during imaging, which is due to moderate photobleaching and/or photochromism of the fluorescence proteins. Because of a significantly faster decline of the FRET signal compared to that of the respective cp173-mEGFP fluorescence, the ratio FRET/GFP of D1GO-Cam (Figure 1C) also moderately decreased over time. This phenomenon might be partially due to cp173-mEGFP (donor) bleed through into the acceptor channel. However, using cells expressing cp173-mEGFP (donor) alone showed only 11.2±4.1% (n = 18) of the donor fluorescence in the respective FRET channel (Figure S1A and S1B). Photobleaching and/or photochromism also occurs if CFP/YFP containing cameleons are used [26], [27] and can be corrected by determining respective bleaching functions (Figures 1B and 1C). A comparison of the photobleaching between D1GO-cam and the CFP/YFP based D3cpv [21] showed, however, clear differences of the kinetics (Figure S1C). While the ratio of FRET/GFP of D1GO-Cam decreased continuously over time with the same slow kinetic, the ratio of FRET/CFP of D3cpv showed initially a fast pronounced decline, probably pointing to direct excitation of the acceptor of D3cpv in the setup used (Figure S1C). Nevertheless, cell stimulation with the Ca^2+^ mobilizing agonist histamine induced fast changes of the respective fluorescence signal (i.e. decrease of the cp173-mEGFP donor fluorescence signal and respective increase of FRET signal), indicating proper functioning of D1GO-Cam as a ratiometric red-shifted genetically encoded Ca^2+^ sensor in intact cells (Figures 1B and 1C). The sensor exhibited a good signal-to noise-ratio, which allowed the recordings of small Ca^2+^ oscillations within HeLa cells in response to the physiological agonist histamine (Figures 1B and 1C).

Next we compared D1GO-Cam with the chemical Ca^2+^ indicator fura-2 in ionomycine treated HeLa cells at low Ca^2+^ concentrations ranging from 30 nM to 2 µM (Figures 1D and 1E). These experiments revealed that D1GO-Cam is suitable to measure Ca^2+^ signals from the nM to the µM range. Using permeabilized HeLa cells the dissociation constant (K_d_) of D1GO-Cam was found to be 1.53 µM (1.32 µM –1.78 µM) at 25°C and pH 7.25 (Figure 1F).

### Co-Imaging of D1GO-Cam with Fura-2

**Figure 2:**
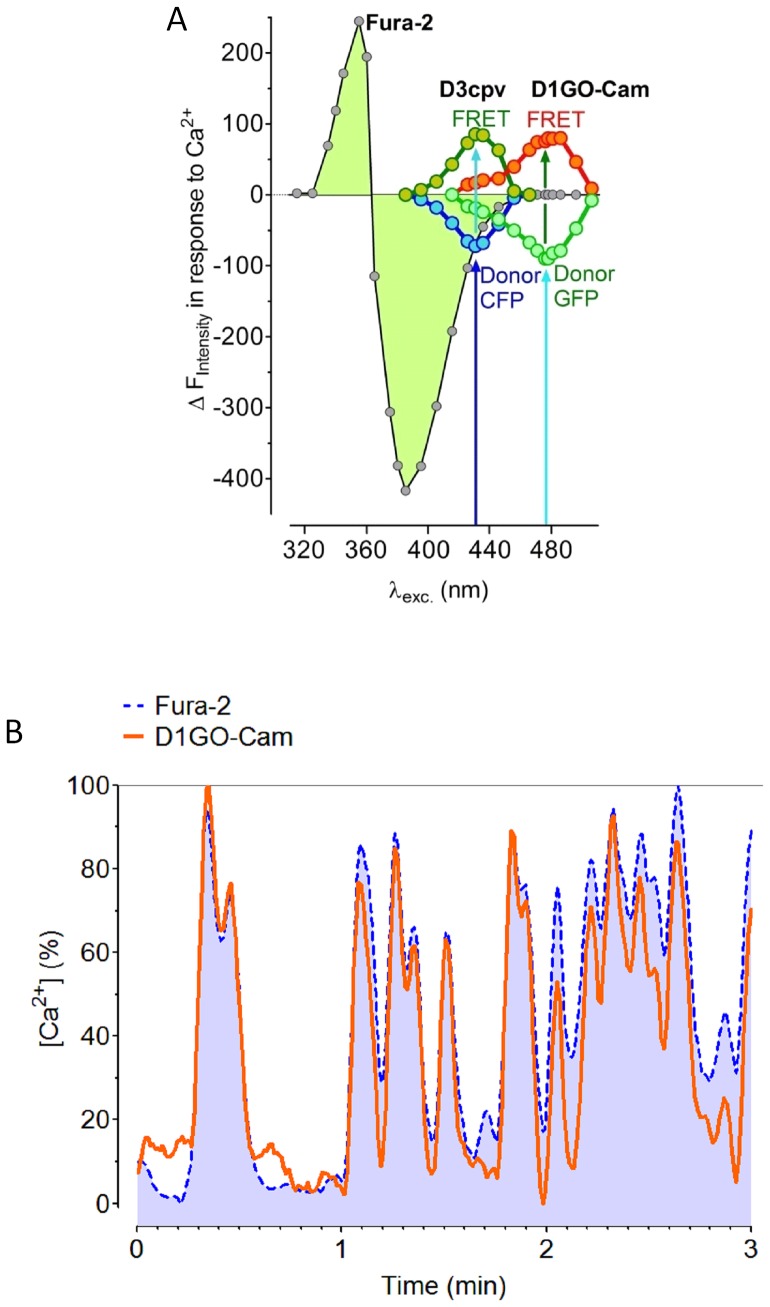
Determination of spectral overlaps between fura-2 and cameleons. (***A***) Spectral overlaps between fura-2 and cameleons are demonstrated by plotting the Ca^2+^ induced changes of the fluorescence intensities at different excitation wavelength ranging from 315 nm –525 nm. Spectral scans were performed with Ea.hy926 cells on a digital wide field fluorescence microscope with an exposure time of 100 ms at each wavelength. Cells were either loaded with fura-2 or transiently transfected with D3cpv or D1GO-Cam. Emissions from the individual excitation wavelengths were taken at 480 nm (CFP of D3cpv), 510 nm (Fura-2 or GFP from D1GO-Cam), 535 nm (FRET of D3cpv), and 560 nm (FRET of D1GO-Cam), respectively. During the scans cells were stimulated with 10 µM ionomycin in the presence of 2 mM Ca^2+^ to induce strong changes of the fluorescence of the Ca^2+^ sensitive probes. (***B***) Representative glucose-induced oscillations of cytosolic Ca^2+^ within same individual INS-1 cells measured simultaneously with fura-2 (dotted blue line) and D1GO-Cam (continuous orange line). Curves are presented in percentage of the respective maximal delta ratio value of the fura-2 signal or the D1GO-Cam signal, respectively.

The actual purpose of the development of a red-shifted cameleon was to minimize fluorescence crosstalk with the ultraviolet excitable chemical Ca^2+^ indicator, fura-2, which should improve the usability of both Ca^2+^ sensors to simultaneously record [Ca^2+^]_cyto_ and [Ca^2+^]_mito_ within one given cell. For testing Ca^2+^ sensitive spectral overlaps between fura-2 and cameleons spectral excitation-scans from 315 nm to 525 nm were performed using cells that were loaded with fura-2/AM or transfected with either the CFP/YFP-based D3cpv or the red-shifted D1GO-Cam. Saturation by Ca^2+^ was achieved by adding ionomycin in the presence of 2 mM extracellular Ca^2+^. These experiments revealed that the fluorescence of fura-2 still significantly declined in response to Ca^2+^ if the chemical Ca^2+^ sensor was excited at 435 nm, which was the optimal excitation wavelength for imaging Ca^2+^ signals with the cameleon D3cpv (Figure 2A). Notably, the fura-2 fluorescence was clearly detectable in both the CFP and the respective FRET channel of the cameleon D3cpv. In contrast, the cross-talk between the fluorescence of fura-2 and the red-shifted ratiometric cameleon, D1GO-Cam, was negligible. D1GO-Cam showed maximal Ca^2+^-induced changes if the probe was excited at 477 nm, which was out of the range to generate significant fura-2 fluorescence (Figure 2A).

Next we imaged cytosolic Ca^2+^ oscillation in the pancreatic *beta-*cell line INS-1 832/13 with both fura-2 and D1GO-Cam simultaneously. As shown in figure 2B the complex patterns of oscillatory changes of [Ca^2+^]_cyto_ in individual fura-2/AM loaded *beta-*cells could be perfectly resolved with D1GO-Cam also. Only small variations of the amplitudes between signals of fura-2 and D1GO-Cam were observed, indicating the suitability of the red-shifted cameleon to resolve such cytosolic Ca^2+^ signals (Figure 2B).

### Co-Imaging of the Mitochondrial Targeted 4mtD1GO-Cam with Fura-2

**Figure 3:**
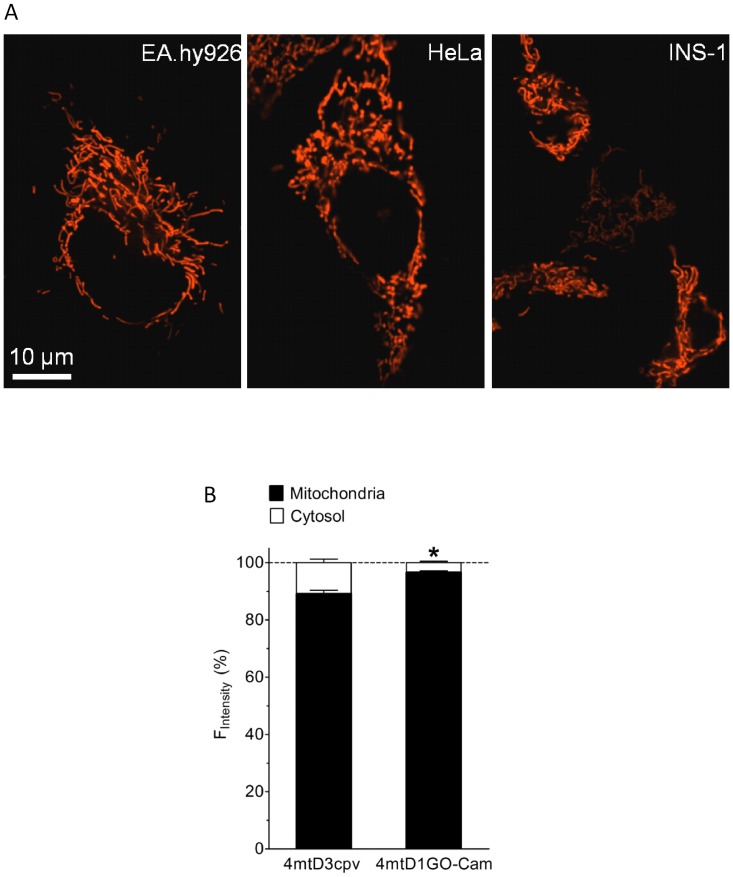
Mitochondrial targeting of 4mtD1GO-Cam. (***A***) Mitochondrial targeting of 4mtD1GO-Cam was visualized on an array confocal laser scanning microscope in Ea.hy926, HeLa, and INS-1 cells. (***B***) The efficiency of mitochondrial targeting of the cameleons 4mtD3cpv and 4mtD1GO-Cam was tested in HeLa cells. The proportion of mitochondrial fluorescence was calculated from both sensors in percentage to that of respective mis-targeted cytosolic fluorescence in individual HeLa cells expressing either 4mtD3cpv (n = 23) or 4mtD1GO-Cam (n = 41). *P<0.05 vs. 4mtD3cpv.

In analogy to the CFP/YFP-based cameleons [21], [28], the mitochondrial signal sequence from subunit VIII of human cytochrome C oxidase (COX VIII) were fused four times in tandem (4mt) at the 5′-end of the D1GO-Cam, in order to ensure successful targeting of the red-shifted cameleon into the mitochondrial matrix. Accordingly, the red-shifted mitochondrial targeted cameleon was named 4mtD1GO-Cam. In all cell types tested the 4mtD1GO-Cam was nicely targeted to mitochondria (Figure 3A). Interestingly, in HeLa cells the 4mtD1GO-Cam showed less mis-targeting compared to the CFP/YFP-based cameleon 4mtD3cpv (Figure 3B).

**Figure 4:**
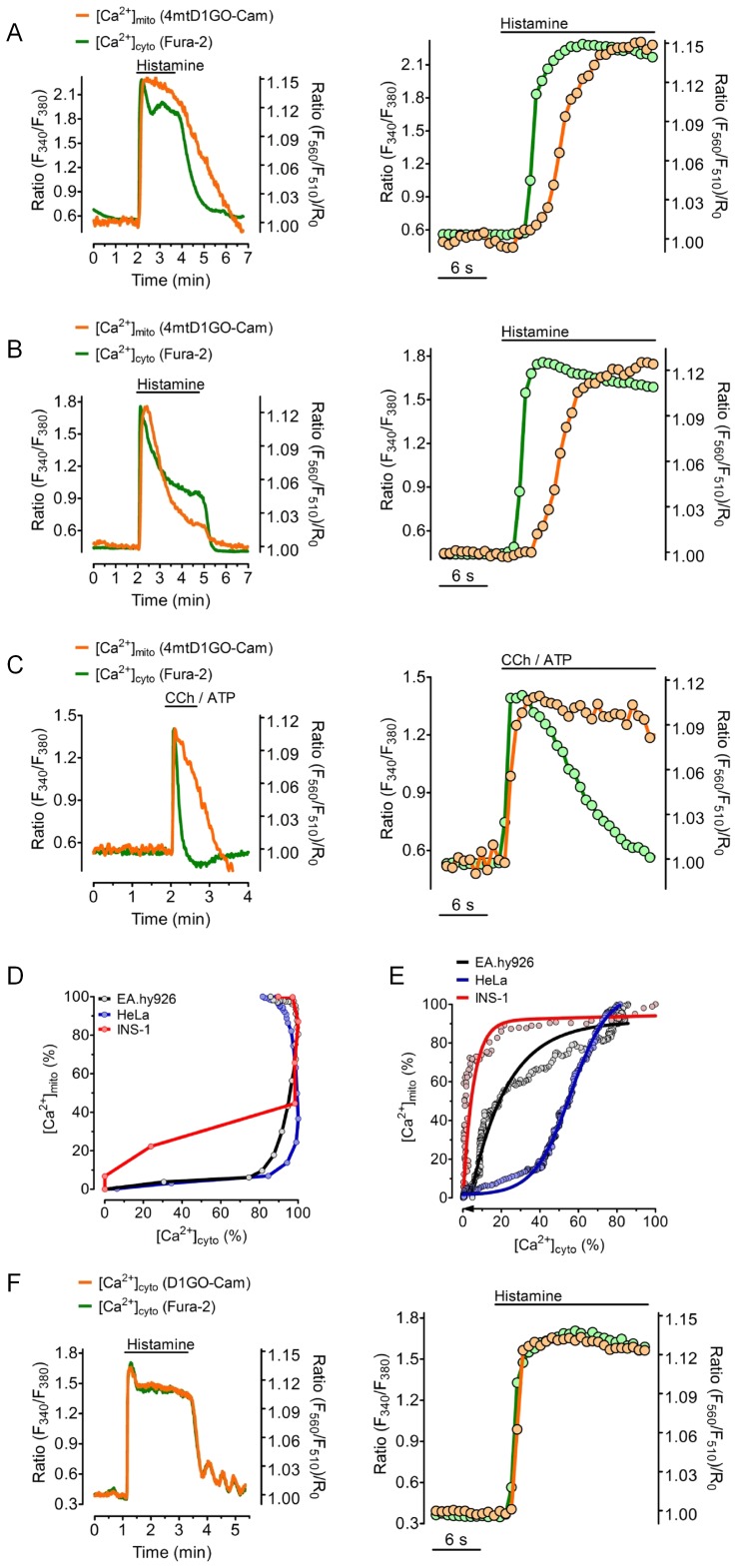
Comparison of cell type specific coupling between [Ca^2+^]_cyto_ and [Ca^2+^]_mito_. (***A–E***) Fura-2/AM loaded cells expression 4mtD1GO-Cam were used to simultaneously record [Ca^2+^]_cyto_ (green traces) and [Ca^2+^]_mito_ (orange traces) in response to cell stimulation with IP_3_-generating agonists. Respective zooms into the rising events are presented on right panels. (***A***) Representative traces of cytosolic and mitochondrial Ca^2+^ signals in Ea.hy926 in response to 100 µM histamine in the presence of 2 mM Ca^2+^. (***B***) Temporal correlation between [Ca^2+^]_cyto_ and [Ca^2+^]_mito_ in HeLa cells in response to 100 µM histamine in the presence of 2 mM Ca^2+^. (***C***) INS-1 cells were treated with a mixture of 100 µM CCh and 100 µM ATP in the absence of Ca^2+^. (***D***) [Ca^2+^]_cyto_ and [Ca^2+^]_mito_ in percentage of the respective maximal increases in response to the treatments shown in panels *A-C* are plotted against each other. Curves are representative for at least 4 independent experiments. (***E***) [Ca^2+^]_cyto_ and [Ca^2+^]_mito_ are plotted against each other for the Ca^2+^ extrusion phases during and after the removal of the IP_3_-generating agonists as indicated in panels *A-C*. (***F***) Representative time course of changes of [Ca^2+^]_cyto_ in EA.hy926 cells in response to 100 µM histamine in the presence of 2 mM Ca^2+^ simultaneously measured with fura-2 and D1GO-Cam. The right panel shows the zoom into the rising event upon cell treatment with histamine.

The suitability of simultaneous recordings of cytosolic and mitochondrial Ca^2+^ concentrations in one given cell were initially tested in the endothelial cell line (EA.hy926) that was stimulated by histamine (Figure 4A). Though a fast increase in free Ca^2+^ in response to the application of the IP_3_-generating agonist was found in both compartments, mitochondrial Ca^2+^ signal was clearly delayed and increased with slower kinetics compared to cytosolic Ca^2+^ elevation (Figure 4A). Notably, mitochondrial Ca^2+^ elevation upon histamine started when the cytosolic Ca^2+^ signal already reached 80% of its maximum (Figure 4D). Moreover, within approximately 2 minutes [Ca^2+^]_cyto_ decreased to basal levels upon the removal of histamine, while the respective mitochondrial Ca^2+^ signal declined considerably slower (Figures 4A and 4E), indicating that in endothelial cells the mitochondrial Ca^2+^ extrusion system acts slower than cytosolic Ca^2+^ clearance. In contrast, in HeLa cells the mitochondrial Ca^2+^ signal was more transient than the respective cytosolic Ca^2+^ elevation in response to histamine (Figures 4B and 4E), evidencing an efficient mitochondrial Ca^2+^ extrusion machinery in this particular cell type. Likewise, in the endothelial cells, histamine-induced rise of [Ca^2+^]_mito_ lagged clearly behind the cytosolic Ca^2+^ signal in HeLa cells (Figures 4B and 4D).

Similar experiments were performed using the pancreatic *beta*-cell line INS-1 832/13 (Figure 4C). In INS-1 832/13 cells, Ca^2+^ was mobilized using a mixture of the two IP_3_-generating agonists carbachol and ATP in the absence of extracellular Ca^2+^. Remarkably, the INS-1 832/13 cells exhibited very different mitochondrial Ca^2+^ kinetics upon cell stimulation compared with our findings in endothelial cells (Figure 4A) and HeLa cells (Figure 4B). In particular while the stimulation of INS-1 832/13 cells led to an almost synchronous transient increase of [Ca^2+^]_mito_ and [Ca^2+^]_cyto_, mitochondrial Ca^2+^ clearance was considerably slower than in the other cell types (Figure 4C and 4E). Although the rise of [Ca^2+^]_mito_ was only moderately slower than the respective cytosolic Ca^2+^ signal, the decrease of [Ca^2+^]_mito_ occurred with a lesser speed compared to that of [Ca^2+^]_cyto_ in this particular cell type (Figures 4B and 4D).

In order to exclude that the differences in the kinetics between [Ca^2+^]_mito_ and [Ca^2+^]_cyto_ are due to differences in the Ca^2+^ affinities and/or the on and off kinetics of the two Ca^2+^ sensors used (i.e. fura-2 and 4mtD1GO-Cam), similar experiments were performed using the non-targeted (i.e. cytosolic) D1GO-Cam simultaneously with fura-2. As shown in figure 4F, the curve obtained with D1GO-Cam was virtually identical with the respective fura-2 signal.

### Temporal Correlations of [Ca^2+^]_mito_ and [Ca^2+^]_cyto_ in Response to Ca^2+^ Entry via L-type Ca^2+^ Channels in INS-1 832/13 Cells

**Figure 5:**
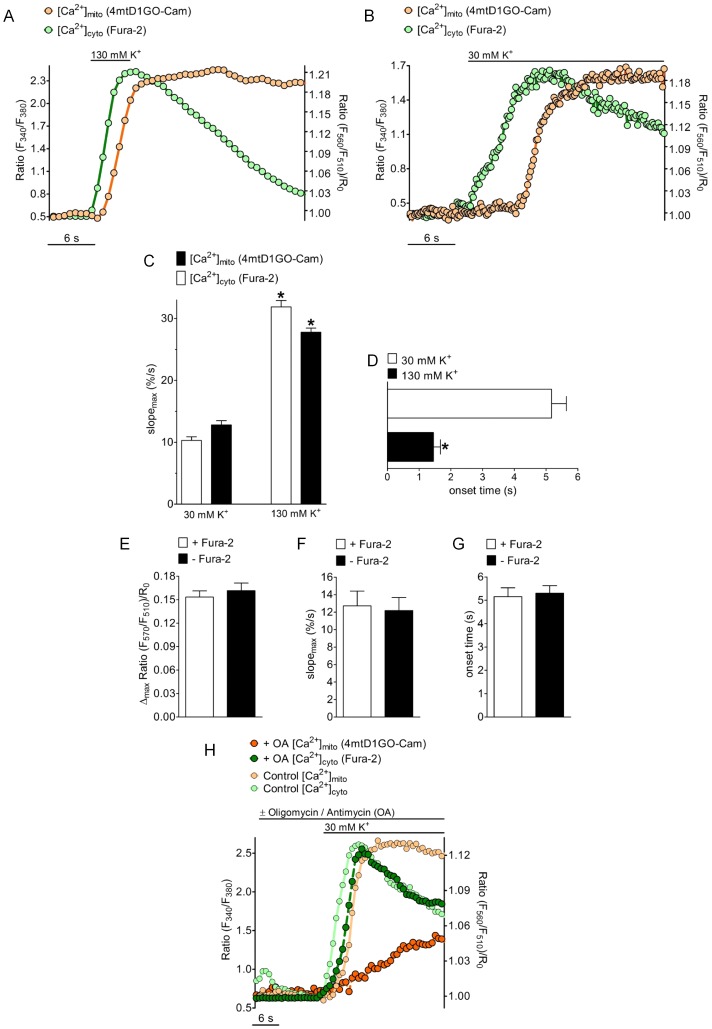
Temporal correlations of [Ca^2+^]_cyto_ and [Ca^2+^]_mito_ in INS-1 cells in response to Ca^2+^ entry via voltage gated Ca^2+^ channels. (*A*) [Ca^2+^]_cyto_ and [Ca^2+^]_mito_ was simultaneously measured in fura-2 loaded INS-1 cells expressing 4mtD1GO-Cam upon cell depolarization with 130 mM K^+^ in the presence of 2 mM Ca^2+^. Rate of data acquisition was 709 ms. (***B***) INS-1 cells were depolarized with 30 mM K^+^ in the presence of 2 mM Ca^2+^. Sampling rate was 189 ms. (***C***) Maximal slopes of [Ca^2+^]_cyto_ (white bars) and [Ca^2+^]_mito_ (black bars) in response to 30 mM K^+^ (right columns, n = 32) and 130 mM K^+^ (left columns, n = 21) were calculated from respective percentage curves. The average maximal effects of respective Ca^2+^ signals induced by cell depolarization with either 30 mM or 130 mM were each defined as 100%. *P<0.05 vs. 30 mM K^+^. (***D***) Statistics of lag times between cytosolic Ca^2+^ rises and mitochondrial Ca^2+^ onsets if cells were stimulated with 30 mM K^+^ (white column, n = 32) and 130 mM K^+^ (black columns, n = 21). *P<0.05 vs. 30 mM K^+^. (***E-G***) Evaluation of the impact of fura-2 on mitochondrial Ca^2+^ signals measured with 4mtD1GO-Cam upon cell treatment with 30 mM K^+^. Columns represent the average of maximal delta ratios (*E*), maximum slopes (*F*), or the lag times between cytosolic Ca^2+^ rises and mitochondrial Ca^2+^ onsets (*G*) of ratio signals from 4mtD1GO-Cam in cells loaded with fura-2/AM (white columns, n = 14) or in the absence of fura-2 (black columns, n = 13). (***H***) Time course of simultaneously measured [Ca^2+^]_cyto_ and [Ca^2+^]_mito_ in individual single INS-1 cells stimulated with 30 mM K^+^ in the presence (*dark curves*) or absence (*light curves*) of 2 µM oligomycin and 10 µM antimycin A. Curves are representative for at least 4 independent experiments.

Subsequently, the temporal correlation between cytosolic and mitochondrial Ca^2+^ elevation upon stimulation of L-type Ca^2+^ channels was tested. Therefore, the excitable INS-1 832/13 cells were depolarized by elevated extracellular K^+^ concentration ([K^+^]_ex_) resulting in Ca^2+^ entry via voltage-gated Ca^2+^ channels [29], [30]. When [K^+^]_ex_ was briefly increased from 5 mM to 130 mM the fura-2 signal transiently rose, while the respective changes in [Ca^2+^]_mito_ were significantly delayed and remained elevated despite the decrease in [Ca^2+^]_cyto_ (Figure 5A). The slopes of rise in [Ca^2+^]_mito_ and [Ca^2+^]_cyto_ were almost identical whereas [Ca^2+^]_cyto_ dropped much faster than the respective mitochondrial Ca^2+^ signal, pointing again to a slower mode of Ca^2+^ extrusion from mitochondria in INS-1 832/13 cells. If cells were moderately depolarized with 30 mM K^+^, [Ca^2+^]_cyto_ increased slowly and the rise of the respective mitochondrial Ca^2+^ was more pronounced (Figures 5B). Strong depolarization with 130 mM K^+^ increased the speed of [Ca^2+^]_mito_ and [Ca^2+^]_cyto_ elevation by about 3-fold (Figure 5C), while the delay between mitochondrial and cytosolic Ca^2+^ signals was reduced by almost 80% (Figure 5D). Notably, the delay between mitochondrial and cytosolic Ca^2+^ signals under these conditions was independent of the sampling rate of image acquisition. These experiments indicated a clear correlation between the kinetics of [Ca^2+^]_cyto_ rise and the temporal delay of the respective mitochondrial Ca^2+^ signals.

In order to test whether or not the differences in the kinetics and temporal patterns between cytosolic and mitochondrial Ca^2+^ signals were due to Ca^2+^ buffering by the chemical Ca^2+^ indicator fura-2, respective experiments were also performed in cells expressing the red-shifted cameleon, 4mtD1GO-Cam, which were not loaded with fura-2/AM. These experiments, however, revealed that neither the amplitude (Figure 5E), nor the slope (Figure 5F), nor the time between cell stimulation until the increase of [Ca^2+^]_mito_ (Figure 5G) was affected by fura-2.

Furthermore, the impact of mitochondrial membrane potential on cytosolic and mitochondrial Ca^2+^ signals was tested in INS-1 832/13 cells in response to cell stimulated with 30 mM K^+^. Therefore mitochondrial membrane potential was collapsed with oligomycin and antimycin A. Under such conditions, the cytosolic Ca^2+^ rise in response to K^+^ was only minimally affected, whereas the respective mitochondrial Ca^2+^ signal was strongly attenuated (Figure 5H).

**Figure 6:**
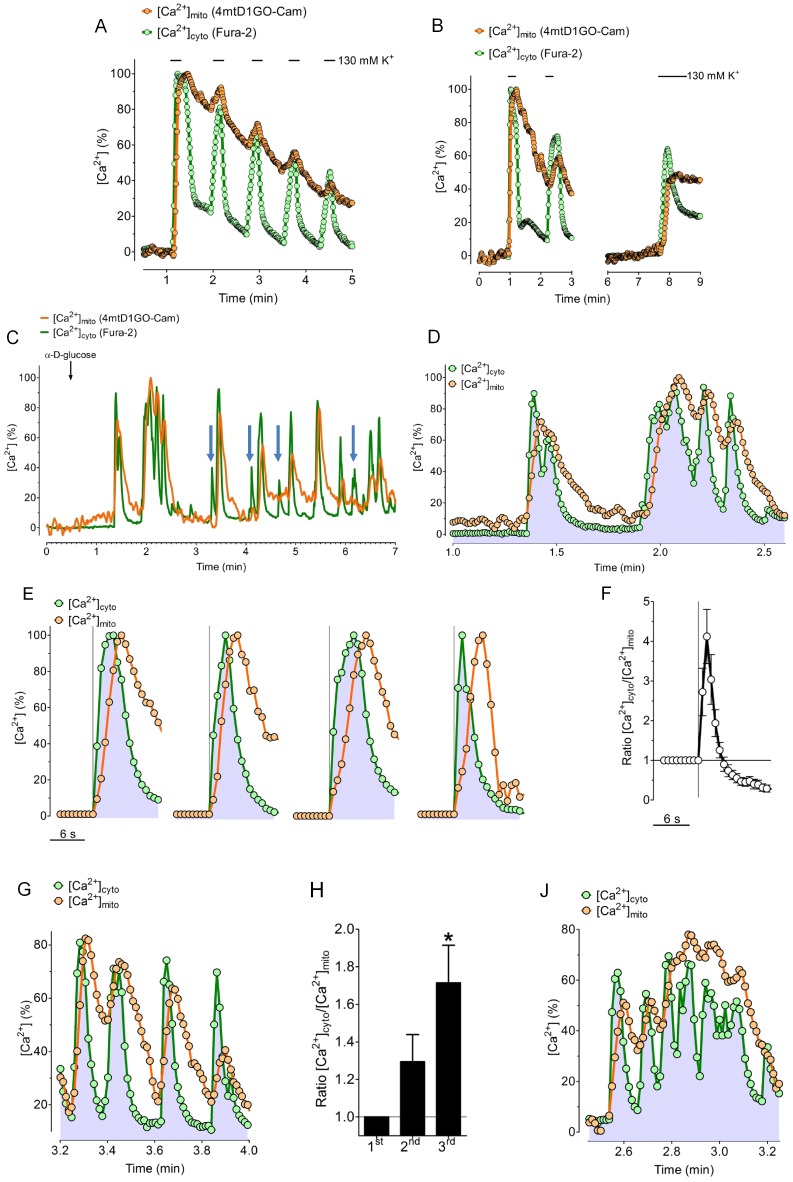
Correlation of repetitive cytosolic and mitochondrial Ca^2+^ transients and glucose-induced Ca^2+^ oscillations in INS-1 cells. (*A*) Fura-2/AM loaded INS-1 cells expressing 4mtD1GO-Cam were repetitively stimulated with short pulses of 130 mM K^+^. The time course of both, [Ca^2+^]_cyto_ (green trace) and [Ca^2+^]_mito_ (orange trace) is plotted as representative curves. (***B***) Representative curves demonstrating [Ca^2+^]_cyto_ and [Ca^2+^]_mito_ of single individual INS-1 cells that were 3 times treated with pulses of high K^+^, with a longer recovery time between the second and third addition of 130 mM K^+^. (***C***) Temporal correlation between [Ca^2+^]_cyto_ and [Ca^2+^]_mito_ of glucose (16 mM) induced Ca^2+^ oscillations in a single individual INS-1 cell. [Ca^2+^]_cyto_ and [Ca^2+^]_mito_ were measured simultaneously using fura-2/AM loaded cells expressing 4mtD1GO-Cam. Blue arrows between minute 3 and 7 indicate clear cytosolic Ca^2+^ signals that were not transferred into mitochondria. (D) Zoom into a typical set of glucose induced Ca^2+^ oscillations in INS-1 cells from the curves presented in panel C. (E) Temporal correlations between [Ca^2+^]_cyto_ (green trace) and [Ca^2+^]_mito_ (orange trace) of single isolated Ca^2+^ transients of INS-1 cells in response to 16 mM glucose from 3 independent experiments. (F) Statistical evaluation of the temporal correlation between [Ca^2+^]_cyto_ and [Ca^2+^]_mito_ of the single isolated Ca^2+^ transients shown in panel A by calculating the average ratio of [Ca^2+^]_cyto_/[Ca^2+^]_mito_ (n = 8). (G) Representative traces of [Ca^2+^]_cyto_ and [Ca^2+^]_mito_ of fast subsequent cytosolic Ca^2+^ transients of INS-1 cells in response to 16 mM glucose. (H) Statistical evaluation of the temporal correlation between [Ca^2+^]_cyto_ and [Ca^2+^]_mito_ of fast subsequent Ca^2+^ transients by calculating the maximal ratios of [Ca^2+^]_cyto_/[Ca^2+^]_mito_ of respective subsequent signals from 3 independent experiments (n = 3). (J) Representative traces of [Ca^2+^]_cyto_ and [Ca^2+^]_mito_ of complex glucose induced Ca^2+^ signal clusters in INS-1 cells.

The correlation between [Ca^2+^]_mito_ and [Ca^2+^]_cyto_ was further studied using repetitive stimulations of INS-1 832/13 cells with short pulses of 130 mM K^+^ (Figures 6A and 6B). Under these conditions each pulse of K^+^ transiently increased [Ca^2+^]_cyto_, whereas the amplitudes of the successive cytosolic Ca^2+^ rises decreased with each K^+^ addition. The cytosolic Ca^2+^ clearance after each K^+^ pulse was, however, very efficient and decreased [Ca^2+^]_cyto_ back to basal levels between the individual K^+^ additions (Figure 6A). In contrast, [Ca^2+^]_mito_ instantly followed the cytosolic Ca^2+^ rise but only slowly decreased after the removal of high K^+^. Notably, successive K^+^ additions triggered only small peaks of [Ca^2+^]_mito_ at elevated levels of [Ca^2+^]_mito_ (Figure 6A). The [Ca^2+^]_mito_ response to cell depolarization with high K^+^ also displayed a time-dependent recovery. Already after a recovery period of 5 minutes, in which [Ca^2+^]_mito_ levels were sufficiently lowered, a further addition of high K^+^ triggered a pronounced increase of [Ca^2+^]_mito_ again (Figure 6B).

To study the relationship between [Ca^2+^]_mito_ and [Ca^2+^]_cyto_ on the single cell level is almost impossible, if both signals are not measured simultaneously in individual cells, which exhibit broad variations in their Ca^2+^ responses to a certain Ca^2+^ mobilizing stimulus. The complex Ca^2+^ oscillations in pancreatic *beta-*cells in response to enhanced glucose metabolism is a good example for a physiological cellular Ca^2+^ response that shows large heterogeneities regarding the amplitudes and frequencies of oscillatory Ca^2+^ signals among individual cells. Accordingly, we studied the correlation of [Ca^2+^]_mito_ and [Ca^2+^]_cyto_ in individual INS-1 cells in response to D-glucose by imaging the red-shifted cameleon, 4mtDIGO-Cam, and fura-2 signals simultaneously (Figures 6C and 6D). This approach unveiled several interesting aspects about the dependency of mitochondrial Ca^2+^ signals on the amplitude and frequency of cytosolic Ca^2+^ spikes: *first*, it was recognized that small cytosolic Ca^2+^ signals are not transferred into mitochondria, pointing to a certain threshold of [Ca^2+^]_cyto_ necessary to activate mitochondrial Ca^2+^ uptake in INS-1 832/13 cells. *Second*, if, however, cytosolic Ca^2+^ elevation reaches the threshold, the rise in [Ca^2+^]_mito_ starts instantly and rises with slightly slower rate (Figure 6D). *Third*, in line with the lag of mitochondrial Ca^2+^ clearance that was observed upon repetitive cell stimulation with high K^+^, under conditions of D-glucose-triggered cytosolic Ca^2+^ spiking, the decrease in [Ca^2+^]_mito_ was clearly slower and delayed compared to the respective oscillatory cytosolic Ca^2+^ signals (Figures 6C and 6D). Consequently, rapid cytosolic Ca^2+^ spiking was not transferred one-to-one into mitochondria but resulted in a long lasting elevation of [Ca^2+^]_mito_ (Figure 6D).

Basically, different Ca^2+^ events in response to high D-glucose with distinct correlations between [Ca^2+^]_mito_ and [Ca^2+^]_cyto_ were observed. Isolated strong cytosolic Ca^2+^ transients were immediately transferred into mitochondria (Figure 6E). Due to a slower kinetic of respective mitochondrial Ca^2+^ rises the ratio of [Ca^2+^]_cyto_/[Ca^2+^]_mito_ of such signals increased transiently (Figure 6F). Fast subsequent cytosolic Ca^2+^ spikes (Figure 6G) were associated with an inactivation of mitochondrial Ca^2+^ uptake with time. Consequently the ratio of [Ca^2+^]_cyto_/[Ca^2+^]_mito_ under such conditions continuously increased with time (Figure 6H). However, INS-1 832/13 cells also showed complex patterns of Ca^2+^ signals in response to high D-glucose (Figure 6J), from which the temporal correlation between [Ca^2+^]_mito_ and [Ca^2+^]_cyto_ was determined by both an inactivation of mitochondrial Ca^2+^ uptake and the slow Ca^2+^ extrusion capacity of mitochondria.

## Discussion

The new red-shifted cameleon, 4mtD1GO-Cam, we have introduced in this study emerged as a suitable tool for measuring mitochondrial Ca^2+^ signals simultaneously with fura-2 in individual cells. Using this technique we were able to precisely correlate [Ca^2+^]_mito_ with [Ca^2+^]_cyto_ in response to different modes of Ca^2+^ mobilization on the single cell level in different cell types.

### Conceptual Design and Characteristics of a Novel Red-shifted Cameleon

Exchanging the most used FRET pair CFP/YFP in cameleons by GFP (cp173-mEGFP) and OFP (mKO_κ_), respectively, made the novel red-shifted genetically encoded Ca^2+^ sensor fully compatible with fura-2 (Figure 2A). The chemical Ca^2+^ indicator fura-2 is the most popular sensor for monitoring changes of the intracellular Ca^2+^ concentration in a confident manner. The reliability and usability of fura-2 is based on, low toxicity, good cell loading properties and, importantly, pronounced ratiometric changes of the fluorescence signals in response to Ca^2+^ [22]. However, the combination of fura-2 with CFP/YFP based FRET probes is problematic because of spectral overlaps [31]. With the imaging system used in this study we recognized that particularly the prominent fura-2 fluorescence contaminates CFP and YFP-FRET channels as the violet excitation light of 435 nm still stimulates fluorescence of the chemical Ca^2+^ indicator. Accordingly, red-shifted FRET probes should be better compatible with the ultra violet (UV) -excitable fura-2. Miyawaki and colleagues constructed several red-shifted cameleons using a red fluorescent protein from a *Discosoma* species (DsRed) as a FRET acceptor [32]. However, because of the broad excitation spectrum of DsRed and the formation of DsRed aggregates, these constructs showed spectral and structural complexities and are, thus, rarely used as Ca^2+^ sensors. The property of DsRed as a FRET acceptor was improved by using a tandem dimer mutant of this fluorescent protein (TDimer2), which was used together with an enhanced GFP (EGFP) to construct a red shifted cameleon [23]. Probably because of the bulkiness of this cameleon containing EGFP and TDimer2 its targeting to mitochondria is difficult and so far this probe has been exclusively used to measure [Ca^2+^]_cyto_. A variety of FRET pairs have been tested for the development of genetically encoded FRET probes, including far red-shifted ones based on orange and red fluorescent proteins [33]. Red-shifted FRET based sensors were recently developed to measure the translocation of annexin A4 [34], caspase-3 activity [35], and changes of the plasma membrane voltage [36], while to our knowledge such FRET pairs have not been used for the construction of red-shifted cameleons so far. Notably, in a recent study a genetically encoded red-shifted ATP sensor that is based on FRET between cp173-mEGFP and the orange fluorescent variant mKO_κ_ was successfully used in combination with fura-2 to correlate [Ca^2+^]_cyto_ with changes of cytosolic or mitochondrial ATP levels [27]. On account of this we designed the cloning of an analog red-shifted cameleon, which we could target effectively to mitochondria (Figures 3A and 3B) by adding the 4mt sequence [21].

The CFP/YFP-based cameleons were improved by engineering CaM and M13, which minimized the interaction with endogenous CaM and increased the range of the measurable Ca^2+^ concentrations. As a result, cameleons with different designs (D) and Ca^2+^ affinities have been developed: D1 with two dissociation constants (K_d_s) of 0.6 µM and 56.5 µM appropriate to measure Ca^2+^ ranging from <1 µM up to >300 µM, D2 with K_d_s ranging from 0.1 to 7.7 µM, D3 with a K_d_ of 0.8 µM, and D4 with a K_d_ of 49.7 µM [24], [37], [38]. In addition both the dynamic range and the pH stability of cameleons were improved by replacing YFP by citrine or circularly permutated venus (cpv) [21]. We decided to generate a red-shifted cameleon containing D1, which should allow monitoring Ca^2+^ over a wide range of Ca^2+^ concentrations. Surprisingly, the Ca^2+^ affinity of the D1GO-Cam was enhanced compared to the reported K_d_s of the respective CFP/YFP based cameleon, D1cpv. Particularly, the D1 containing red-shifted cameleon exhibited just one K_d_ of 1.53 µM and was already saturated at Ca^2+^ concentrations >100 µM, when we calibrated the sensor in situ (Figure 1F). The reported K_d_s of the D1cpv were obtained in vitro [21], which, however, cannot explain the clear discrepancy between the Ca^2+^ affinities. Hence, we speculate that the shift towards a higher Ca^2+^ sensitivity in D1GO-Cam is based on the exchange of the fluorescent proteins. It has been recognized that the interaction between fluorescent proteins impacts on the K_d_s of FRET-based sensors [23]. Nevertheless, the exchange of CFP/YFP to cp173-mEGFP/mKO_κ_ in a genetically encoded ATP sensor did not increase, but reduced the affinity to ATP [21]. Accordingly, we rather expected a diminished Ca^2+^ affinity of the red-shifted cameleon compared to the CFP/YFP based Ca^2+^ probe. Moreover, another explanation for the shift of the K_d_ of the red-shifted cameleon might be the orientation of the fluorophores, as the sequential arrangement of the donor and acceptor fluorescent proteins in D1GO-Cam (Figure 1A) is *vice versa* compared with the classical cameleons [16], [24], [39]. A more consistent explanation for the differences in the K_d_s between D1GO-Cam and D1ER might be based on the different dimerization properties of the different fluorescent proteins. CFP and YFP of D1ER might exhibit weak dimerization, while cp173-mEGFP and mKO_κ_ do not. Accordingly, a conformational change of the CaM/M13 domain in D1ER might facilitate dimerization of the fluorescent proteins, which explains the higher K_d_ of approximately 60 µM.

### Spatiotemporal Correlations between [Ca^2+^]_mito_ and [Ca^2+^]_cyto_

The red-shifted cameleon could be nicely targeted to mitochondria in different cell types (Figure 3A). Although the same targeting sequence (4mt) for mitochondrial targeting was used, 4mtD1GO-Cam showed less mis-targeting compared to the respective 4mtD3cpv (Figure 3B). We could not elaborate the reason for this difference. However, this finding should be considered with caution, as the autofluorescence of cells might be higher within the 4mtD3cpv excitation/emission range.

Our measurements show that the accumulation of Ca^2+^ within mitochondria clearly lags behind cytosolic Ca^2+^ signals, particularly if [Ca^2+^]_cyto_ rises rather slowly (Figures 4A, 4B and 5A, 5B, 5D). This finding is in line with early studies indicating that the mitochondrial Ca^2+^ uniporter, the main route for mitochondrial Ca^2+^ uptake, has a pretty low affinity for Ca^2+^ [40]. Similar findings were obtained using confocal imaging of Rhod-2-loaded HeLa cells, in which [Ca^2+^]_mito_ and [Ca^2+^]_cyto_ was also assessed simultaneously [41]. In this study it was calculated that [Ca^2+^]_cyto_ increases to approximately 400–600 nM before mitochondria start to accumulate Ca^2+^ efficiently. Accordingly, mitochondrial Ca^2+^ uptake requires a certain threshold of [Ca^2+^]_cyto_ that is necessary to stimulate the low affinity of the mitochondrial Ca^2+^ uptake machinery. Otherwise it has been proposed that high Ca^2+^ microdomains are formed between the ER and mitochondria, which allow the activation of mitochondrial Ca^2+^ uptake [42]. Indeed, the existence of such local Ca^2+^ microdomains between the organelles during cell stimulations with an IP_3_-generating agonist was recently experimentally proved [43]. In an elegant study using a rapamycin inducible linker with different lengths it was indicated that the area and gap width of ER-mitochondria junctions determine the local Ca^2+^ microdomains between the organelles [44]. Although these studies indicated that high Ca^2+^ microdomains on the surface of mitochondria existed in a heterogeneous manner in same cells, little is known about their temporal pattern. Based on the lag between rises of cytosolic and mitochondrial Ca^2+^ signals obtained in this study, it is tempting to speculate that the formation of effective Ca^2+^ microdomains in the vicinity of sites of mitochondrial Ca^2+^ uptake is a rather slow process. This is feasible considering the possibility that Ca^2+^ itself controls the organization of ER-mitochondria junctions via the regulation of the morphology and dynamics of the organelles [45], [46]. In addition the activation or even the formation of sites of mitochondrial Ca^2+^ uptake by Ca^2+^ might be a time consuming process.

Interestingly, the speed of the transfer of cytosolic Ca^2+^ into mitochondria was not just depending on the mode and strength of Ca^2+^ mobilization but also on the cell type used (Figure 4A-E and Figure S1). These findings might as well indicate that mitochondria in different cell types are equipped with different forms and/or amounts of mitochondrial Ca^2+^ channels and antiporters [13], . Indeed, we and others recently found evidences that distinct mitochondrial Ca^2+^ channels are functionally expressed in different cell types using the patch clamp technique on mitoplasts [13], [48]. In line with this finding two distinct mitochondrial Ca^2+^ currents have been identified in mitoplasts from human cardiomyocytes [49]. Nevertheless, the identity of proteins probably forming mitochondrial Ca^2+^ uptake machineries remained elusive until recently. In the last few years different studies indicated that a mitochondrial ryanodine receptor [50], the uncoupling proteins 2 and 3 (UCP2/3) [51], [52], the leucine zipper EF hand-containing transmembrane protein 1 (Letm1) [19], [53], mitochondrial Ca^2+^ uptake 1 (MICU1) [54] and a very recently identified protein, referred to as mitochondrial Ca^2+^ uniporter (MCU) [13], [55], are prospective candidates to catalyze and/or transfer the Ca^2+^ into mitochondria. However, further studies are necessary to understand the exact functional and physiological roles of these proteins for the mitochondrial Ca^2+^ homeostasis in different cell types [23]. The novel approach we have introduced herein to simultaneously measure both, [Ca^2+^]_mito_ and [Ca^2+^]_cyto_ in a ratiometric manner might help to better cope with these tasks.

It has been shown that the content of polyphosphate within mitochondria clearly affects mitochondrial Ca^2+^ signals [56], [57]. Hence, the differences of the speed and capacity of mitochondrial Ca^2+^ uptake in the different cell types we have shown in this study might also point to differences in the polyphosphate content and, thus, the mitochondrial Ca^2+^ buffer capacity of the different cell models used. This is possible particularly because of differences in the rate of ATP generating metabolic pathways within the different cells. In INS-1 832/13 cells most of the ATP is produced by oxidative phosphorylation [58], while in HeLa and EA.hy926 cells ATP is generated primarily by anaerobic glycolysis with a slow rate of oxidative phosphorylation [59], [60].

The kinetics of the export of Ca^2+^ from mitochondria was also diverse in the three different cell lines used in this study (Figure 4A-E). Mitochondria in HeLa cells were highly effective in extruding Ca^2+^ ions (Figure 4B and 4E), while in cells from both the endothelial (Figure 4A) and pancreatic *beta*-cell line (Figure 4C), the kinetics of the clearance of Ca^2+^ from mitochondria was evidently slower than the respective cytosolic Ca^2+^ declines. These findings also indicate that the mitochondrial Ca^2+^ homeostasis is differently controlled in different cell types, which impacts on the characterization and identification of the mitochondrial Ca^2+^ proteome. We obtained results indicating that the red-shifted cameleon actually measures the correct kinetics of mitochondrial Ca^2+^ signals, because the kinetics of the cytosolic Ca^2+^ signals within individual single cells were almost identical independently whether [Ca^2+^]_cyto_ was recorded with fura-2 or D1GO-Cam (Figures 2B and 4F), and second the ratiometric signals of the 4mtD1GO-Cam is hardly pH sensitive as the high pH stability of the cp173-mEGFP/mKO_κ_ FRET pair was already documented [61] and further suggests that the novel red-shifted cameleon is especially suitable to monitor [Ca^2+^]_mito_.

Testing the 4mtD1GO-Cam in fura-2/AM loaded INS-1 832/13 cells revealed several interesting aspects regarding the coupling of mitochondrial Ca^2+^ uptake to cytosolic Ca^2+^ transients in this particular cell type. The decrease of mitochondrial Ca^2+^ loads upon repetitive cell stimulations with high K^+^ (Figures 6A and 6B) points to a Ca^2+^ desensitization of the mitochondrial Ca^2+^ uptake pathway in INS-1 832/13 cells. A Ca^2+^ dependent inactivation of mitochondrial Ca^2+^ uptake in pancreatic *beta*-cells was also found in response to methyl succinate [62]. Moreover, the phenomenon of a complex regulation of the mitochondrial Ca^2+^ uptake pathway(s) by Ca^2+^ was also established in other cell types [29], [40], [63].

The suitability of the novel approach to simultaneously record [Ca^2+^]_mito_ and [Ca^2+^]_cyto_ was finally tested in INS-1 832/13 cells that exhibited complex and heterogeneous Ca^2+^ oscillations in response to D-glucose [64]. In pancreatic *beta*-cells the transfer of Ca^2+^ into mitochondria is of utmost importance as mitochondrial Ca^2+^ sequestration facilitates glucose-induced insulin secretion [3], [65]. Hence, the characterization and identification of the mitochondrial Ca^2+^ homeostasis is highly relevant for better understanding the physiology and pathology of pancreatic *beta*-cells. Our measurements revealed that in contrast to cell stimulation with high K^+^ the glucose-induced robust cytosolic Ca^2+^ signals are instantly transferred into mitochondria in INS-1 832/13 cells (Figures 6C and 6D). This tight Ca^2+^ coupling might point to additional processes, which facilitate the sequestration of Ca^2+^ by mitochondria, if the metabolism of glucose is triggering Ca^2+^ entry via voltage-gated Ca^2+^ channels in pancreatic *beta*-cells. However, the slow rate of mitochondrial Ca^2+^ extrusion and the desensitization of mitochondrial Ca^2+^ uptake in response to high K^+^ were also obvious in the oscillatory signals triggered by glucose (Figures 6C and 6D). These findings are in line with a recent report in which Ca^2+^ signals were measured with mitochondrial targeted pericam and fura red in INS-1 cells [66].

### Conclusion

The novel red-shifted genetically encoded Ca^2+^ probe developed in this study is a suitable ratiometric Ca^2+^ indicator, which can be used in combination with fura-2 to correlate [Ca^2+^]_cyto_ with localized Ca^2+^ signals on the single cell level. The use of this novel probe can be easily performed on conventional wide field imaging systems and produces reliable Ca^2+^ measurements in a ratiometric manner appropriate to simultaneously quantify global cytosolic and organelle Ca^2+^ signals.

## Materials and Methods

### Materials

For cell culture, HAT supplement was purchased at Invitrogen (Vienna, Austria). Other media supplements, RPMI-1640, fetal calf serum (FCS) and all plastic ware were from PAA laboratories (Pasching, Austria). Dulbecco’s modified eagle’s medium (DMEM), histamine dihydrochloride (Histamine), carbamylcholin chloride (Carbachol, CCh), adenosine 5′-triphosphate disodium salt (ATP), oligomycin and antimycin A were obtained from Sigma–Aldrich (Vienna, Austria). Ionomycin (free acid) was from abcamBiochemicals (Cambridge, UK) and fura-2/AM from Teflabs (Texas Fluorescence Labs Inc., Austin, Tx, USA). Transfast™ transfection reagent, restriction enzymes and Taq polymerase used for subcloning were purchased from Promega (Mannheim, Germany). All other chemicals were obtained from Roth (Karlsruhe, Germany).

### Cell Culture, Fura-2/AM Loading and Transfection

In this study all 3 different cell lines used were cultured and transfected in a humidified atmosphere at 37°C and 5% CO_2_. INS-1 832/13 cells (INS-1) were kindly provided by C. B. Newgard (Duke University School of Medicine) and cultured with RPMI-1640 culture medium containing 2 mM L-glutamate and 11.1 mM D-glucose supplemented with 10% FCS, 1 mM sodium pyruvate, 5 µM mercaptoethanol, 100 U/ml penicillin and 100 µg/ml streptomycin. The human umbilical vein endothelial cell line EA.hy926 [24] was cultured in DMEM containing 10% FCS, 100 U/ml penicillin, 100 µg/ml streptomycin and 1% HAT (5 mM hypoxanthin, 20 µM aminopterin and 0.8 mM thymidine). HeLa cells were cultured in the same medium without HAT supplement. For experiments and transfection, cells were grown on 30 mm glass cover slips and transfected at 50–80% confluence with 1.5 µg of plasmid DNA (per 30 mm well) using 4 µg/well TransFast™ transfection reagent in 0.5 ml of serum and antibiotic-free transfection medium. Cells were maintained in the incubator (37°C, 5% CO_2_, 95% air) for 16–20 hours before changing the medium back to normal culture medium. Experiments were performed 24–48 hours after transfection. Cells were loaded at RT with 2 µM fura-2/AM for 20 minutes in a HEPES buffered solution containing in mM: 135 NaCl, KCl, 2 CaCl_2_, 1 MgCl_2_, 1 HEPES, 2.6 NaHCO_3_, 0.44 KH_2_PO_4_, 0.34 Na_2_HPO_4_, 10 D-glucose, 0.1% vitamins, 0.2% essential amino acids and 1% penicillin/streptomycin pH 7.4. Prior to experiments cells were washed and stored in the same HEPES buffered solution without fura-2/AM.

### Design and Construction of D1GO-Cam and 4mtD1GO-Cam

The Ca^2+^ sensitive calmodulin/M13 sequence (D1) from the D1ER probe [23] was amplified via PCR and the internal ClaI restriction site was deleted by silent mutation using the primers as follows: 5′-GGATCGATATGCATGACCAACTGACAGAA-3′, 5′-GGCCATCACCGTCAATATCT-3′, 5′-GGAAGCAGATATTGACGGTG-3′ and 5′-AAGAATTCCATGAGCTCCAGTGCCCCG-3′. D1 was then subcloned via its overhanging ClaI and EcoRI recognition sites into the newly developed red-shifted mitochondrial or cytosolic ATP sensor (4mtGO-ATeam or GO-ATeam) [67] after removing the ATP sensing ε subunit of the bacterial F_o_F_1_-ATP synthase sequence. Accordingly, exchanging the ATP sensing ε subunit to the calmodulin/M13 sequences of D1 resulted in 2 new plasmids coding for Ca^2+^ sensitive probes: the cytosolic D1GO-Cam and the mitochondrial targeted variant, 4mtD1GO-Cam, respectively.

### Experimental Buffers for Ca^2+^ Measurments

Ca^2+^ measurements in Ea.hy926 and HeLa cells were performed by stimulating the cells in a Ca^2+^ containing environment by perfusing the cells in a calcium containing buffer (CB), which was composed of (in mM): 138 NaCl, 5 KCl, 2 CaCl_2_, 1 MgCl_2_, 10 D-glucose and 10 HEPES, pH adjusted to 7.4 with NaOH. Stimulation of INS-1 with IP3-generating agonists was done using 100 µM CCh and 200 µM ATP under Ca^2+^ free conditions in an EGTA containing buffer (EB) that was composed like the CB, but contained 1 mM EGTA instead of 2 mM Ca^2+^. In order to trigger K^+^ induced depolarization in this cell line we used the CB and enhanced the K^+^ concentration from 5 mM to 30 mM or 130 mM K^+^, respectively. The concentration of NaCl was reduced accordingly in high K^+^ buffers to maintain osmolarity. Moreover, the impact of mitochondrial depolarization on [Ca^2+^]_mito_ and [Ca^2+^]_cyto_ was simultaneously observed using 2 µM oligomycin and 10 µM antimycin under this condition. For glucose-stimulated Ca^2+^ measurements INS-1 cells were preincubated in HBSS with (in mM) 114 NaCl, 4.7 KCl, 2.5 CaCl_2,_ 1.2 MgSO_4_, 2 HEPES, 1.2 KH_2_PO_4_, 25 NaHCO_3_, 0.2% bovine serum albumin (BSA) and 3 D-glucose for 30 minutes and then washed and equilibrated in CB (without glucose) for 10–15 minutes before imaging. Glucose-stimulated Ca^2+^ signals were then observed by increasing glucose from 0 mM to 16 mM in CB. For simultaneous measurements cells transfected with one of the GO-cam plasmids were loaded with fura-2/AM as described. Single cells containing both, the fura-2 and the GO-cam biosensor were alternately excited at 340, 380 or 477 nm and emissions were recorded at 510 nm for fura-2 and GFP or at 560 nm for FRET channel.

### Single Cell Ca^2+^ Imaging and Data Acquisition

Co-imaging of fura-2 and the red-shifted cameleons was performed on a digital wide field imaging system, the Till iMIC (Till Photonics Graefelfing, Germany) using a 40× objective (alpha Plan Fluar 40×, Zeiss, Göttingen, Germany). For illumination of fura-2 and the cameleons an ultra fast switching monochromator, the Polychrome V (Till Photonics) was used. Fura-2 was excited alternatively at 340 nm and 380 nm and the red-shifted cameleons were excited at 477 nm, respectively. To avoid contamination of the emission channels with excitation light an excitation filter (E500spuv, Chroma Technology Corp, Rockingham Vermont, USA) and a dichroic filter (495dcxru) were installed. Emission light was simultaneously collected at 510 nm (fura-2 and GFP of GO-Cams) and at 560 nm (FRET-channel of GO-Cams) using a single beam splitter design (Dichrotome, Till Photonics) that was equipped with a dual band emission filter (59004m ET Fitc/Tritc Dual Emitter, Chroma Technology Corp) and a second dichroic filter (560dcxr, Chroma Technology Corp). The light path and spectra for the filters used for the co-imaging experiments are illustrated in figure S2. Images were recorded with a charged-coupled device (CCD) camera (AVT Stringray F145B, Allied Vision Technologies, Stadtroda, Germany). For the data acquisition and the control of the digital fluorescence microscope the live acquisition software version 2.0.0.12 (Till Photonics) was used.

## Supporting Information

Figure S1 **Characterization of spectral properties and photobleaching of the fluorescence proteins cp173-mEGFP and mKO_κ_ of D1GO-Cam, respectively.** (A) Representative images of HeLa cells expressing either D1GO-Cam (upper panel) or cp173-mEGFP alone (middle panel) or mKO_κ_ alone (lower panel) showing the FRET (acceptor) channel (emission at ∼ 560 nm) on the left images and the GFP (donor) channel (emmsion at ∼ 510 nM) on the right images, respectively. The white scale bar in the upper left image represents 20 µm. (B) Statistical analysis of the contribution of the fluorescence signals of the D1GO-Cam (left pairs of columns, n = 10) under resting conditions (i.e. low Ca^2+^ levels) in the FRET (acceptor) channel (left orange column) and the GFP (donor) channel (left green column) and the contribution of the fluorescence signals of cp173-mEGFP alone (right pairs of columns, n = 18) in the FRET (acceptor) channel (right orange column) and the GFP (donor) channel (right green column). (C) Quantitative comparison of the photobleaching of D3cpv (containing the CFP/YFP FRET pair) relative to the D1GO-Cam (containing the GFP/OFP FRET pair). HeLa cells expressing the D3cpv (n = 15) were illuminated with excitation light at 430 nm with an exposure time of 400 ms and a camera binning of 4. Emission light was collected simultaneously at 480 nM (CFP donor fluorescence) and at 535 nm (FRET acceptor fluorescence) using the beam splitter device. With the same settings cells expressing D1GO-Cam (n = 12) were illuminated at 477 nm and emission light was collected at 510 nm (GFP, donor fluorescence) and 560 nm (FRET acceptor fluorescence), respectively. For both sensors the ratio FRET/F_donor_ was plotted over time.
(TIF)

Figure S2 **Imaging setup for the simultaneous recording of fura-2 and the novel red-shifted cameleons.** Schematic representation of the imaging system with light paths of the excitation - (violet  = 340 nm, dark blue  = 380 nm, and light blue 477 nm) and emission light (green  = 510 nM and orange  = 560 nM) and the optical filters used to simultaneously image fura-2 and the novel red-shifted cameleons.
(TIF)
