# Supplementary figures and images for: Spatiotemporal Correlations between Cytosolic and Mitochondrial Ca2+ Signals Using a Novel Red-Shifted Mitochondrial Targeted Cameleon
Source: PLoS One. 2012 Sep 21;7(9):e45917. doi: 10.1371/journal.pone.0045917 (PMC3448721; doi:10.1371/journal.pone.0045917)

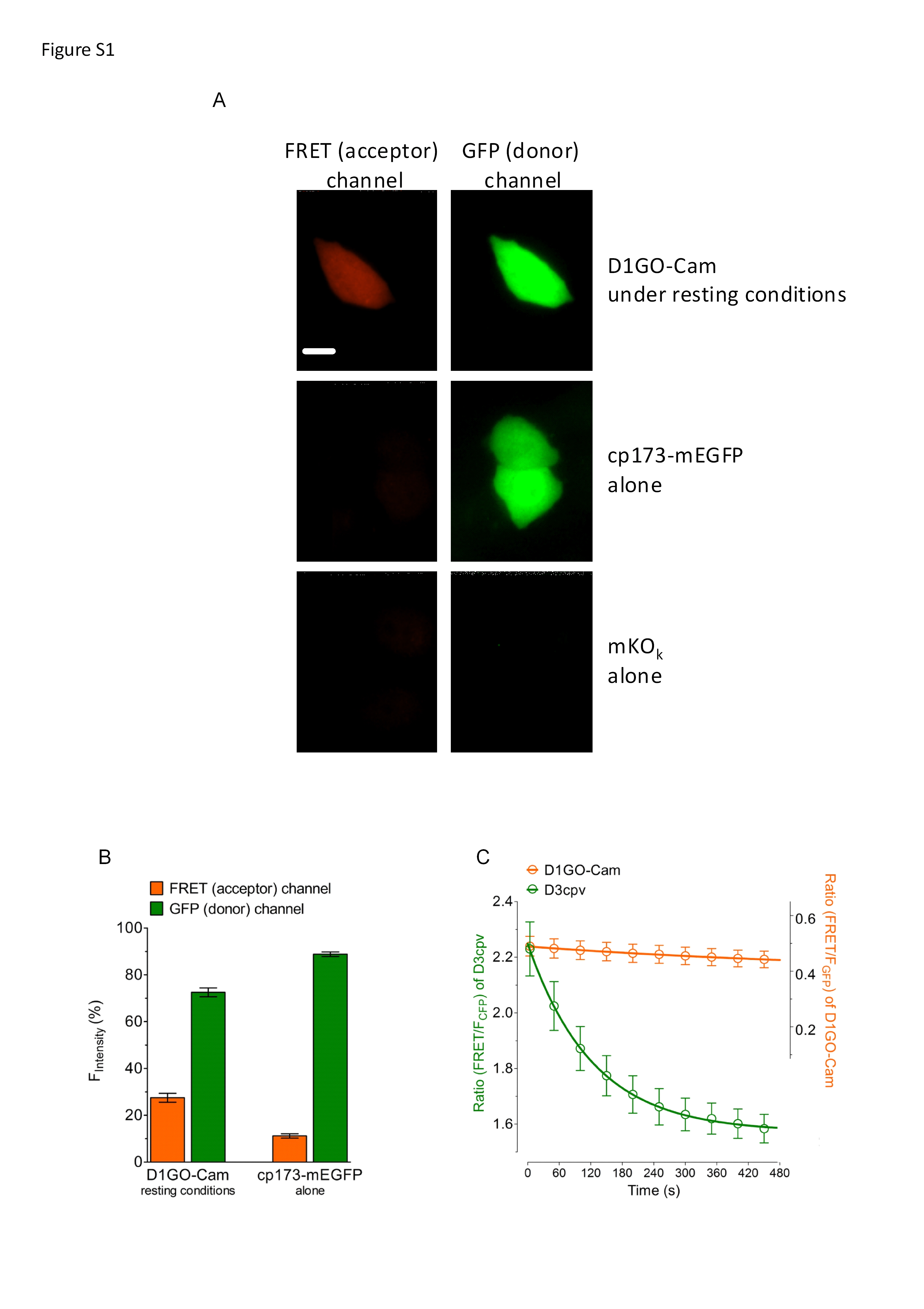

Supplement: Figure S1 — Characterization of spectral properties and photobleaching of the fluorescence proteins cp173-mEGFP and mKOκ of D1GO-Cam, respectively. (A) Representative images of HeLa cells expressing either D1GO-Cam (upper panel) or cp173-mEGFP alone (middle panel) or mKOκ alone (lower panel) showing the FRET (acceptor) channel (emission at ∼ 560 nm) on the left images and the GFP (donor) channel (emmsion at ∼ 510 nM) on the right images, respectively. The white scale bar in the upper left image represents 20 µm. (B) Statistical analysis of the contribution of the fluorescence signals of the D1GO-Cam (left pairs of columns, n = 10) under resting conditions (i.e. low Ca2+ levels) in the FRET (acceptor) channel (left orange column) and the GFP (donor) channel (left green column) and the contribution of the fluorescence signals of cp173-mEGFP alone (right pairs of columns, n = 18) in the FRET (acceptor) channel (right orange column) and the GFP (donor) channel (right green column). (C) Quantitative comparison of the photobleaching of D3cpv (containing the CFP/YFP FRET pair) relative to the D1GO-Cam (containing the GFP/OFP FRET pair). HeLa cells expressing the D3cpv (n = 15) were illuminated with excitation light at 430 nm with an exposure time of 400 ms and a camera binning of 4. Emission light was collected simultaneously at 480 nM (CFP donor fluorescence) and at 535 nm (FRET acceptor fluorescence) using the beam splitter device. With the same settings cells expressing D1GO-Cam (n = 12) were illuminated at 477 nm and emission light was collected at 510 nm (GFP, donor fluorescence) and 560 nm (FRET acceptor fluorescence), respectively. For both sensors the ratio FRET/Fdonor was plotted over time. (TIF) [file pone.0045917.s001.tif]

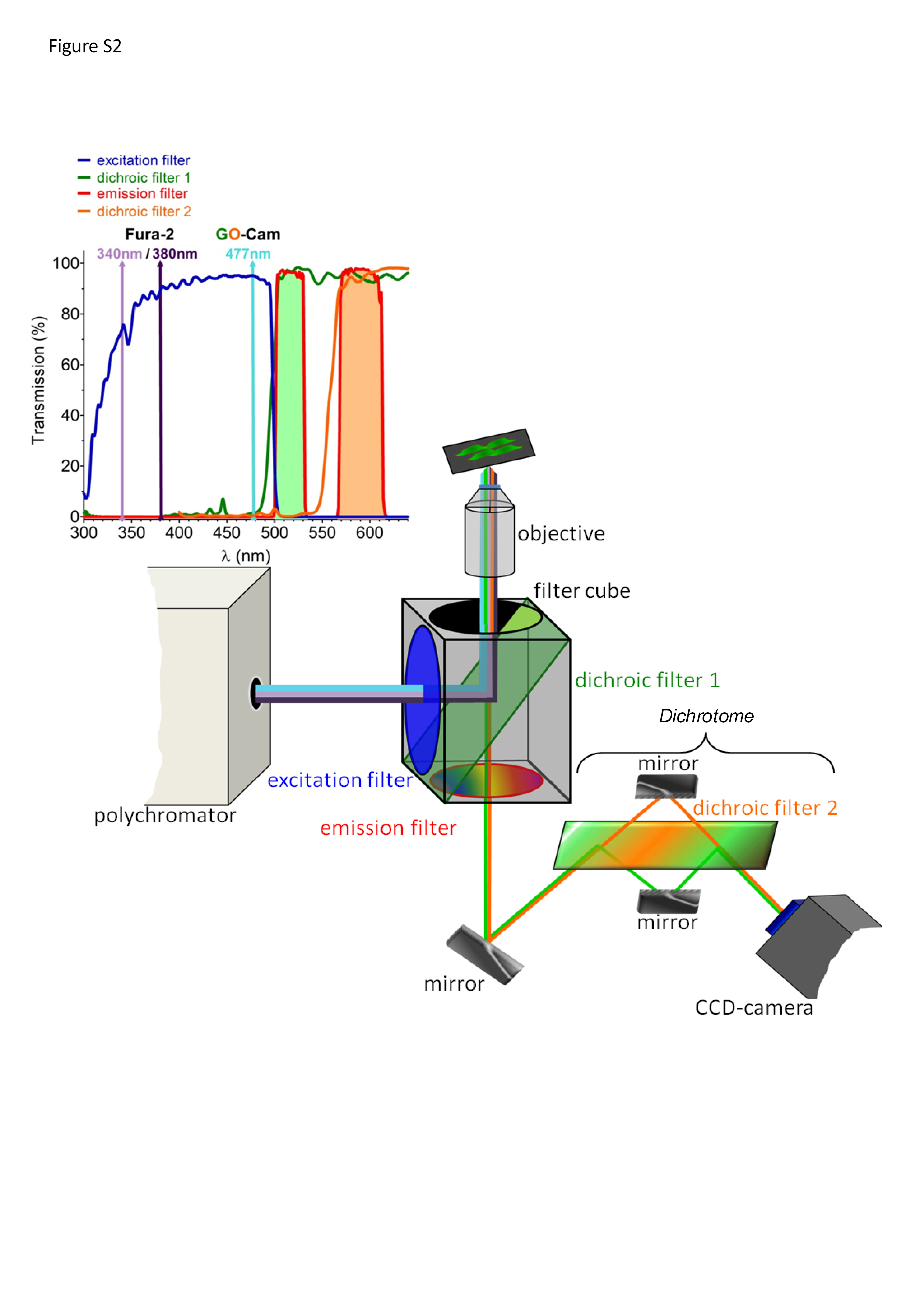

Supplement: Figure S2 — Imaging setup for the simultaneous recording of fura-2 and the novel red-shifted cameleons. Schematic representation of the imaging system with light paths of the excitation - (violet = 340 nm, dark blue = 380 nm, and light blue 477 nm) and emission light (green = 510 nM and orange = 560 nM) and the optical filters used to simultaneously image fura-2 and the novel red-shifted cameleons. (TIF) [file pone.0045917.s002.tif]
